# Automatic selection of partitioning schemes for phylogenetic analyses using iterative *k*-means clustering of site rates

**DOI:** 10.1186/s12862-015-0283-7

**Published:** 2015-02-10

**Authors:** Paul B Frandsen, Brett Calcott, Christoph Mayer, Robert Lanfear

**Affiliations:** Office of Research Information Services, Office of the CIO, Smithsonian Institution, Washington, D.C. USA; Department of Entomology, Rutgers University, New Brunswick, New Jersey USA; School of Life Sciences, Arizona State University, Tempe, AZ USA; Zoologisches Forschungsmuseum Alexander Koenig (ZFMK)/Zentrum für Molekulare Biodiversitätsforschung (ZMB), Bonn, Germany; Ecology Evolution and Genetics, Research School of Biology, Australian National University, Canberra, ACT Australia; National Evolutionary Synthesis Center, Durham, NC USA; Department of Biological Sciences, Macquarie University, Sydney, Australia

**Keywords:** Model selection, Partitioning, Partitionfinder, Phylogenetics, Phylogenomics, K-means, Clustering, Ultra-conserved elements, UCE’s

## Abstract

**Background:**

Model selection is a vital part of most phylogenetic analyses, and accounting for the heterogeneity in evolutionary patterns across sites is particularly important. Mixture models and partitioning are commonly used to account for this variation, and partitioning is the most popular approach. Most current partitioning methods require some *a priori* partitioning scheme to be defined, typically guided by known structural features of the sequences, such as gene boundaries or codon positions. Recent evidence suggests that these *a priori* boundaries often fail to adequately account for variation in rates and patterns of evolution among sites. Furthermore, new phylogenomic datasets such as those assembled from ultra-conserved elements lack obvious structural features on which to define *a priori* partitioning schemes. The upshot is that, for many phylogenetic datasets, partitioned models of molecular evolution may be inadequate, thus limiting the accuracy of downstream phylogenetic analyses.

**Results:**

We present a new algorithm that automatically selects a partitioning scheme via the iterative division of the alignment into subsets of similar sites based on their rates of evolution. We compare this method to existing approaches using a wide range of empirical datasets, and show that it consistently leads to large increases in the fit of partitioned models of molecular evolution when measured using AICc and BIC scores. In doing so, we demonstrate that some related approaches to solving this problem may have been associated with a small but important bias.

**Conclusions:**

Our method provides an alternative to traditional approaches to partitioning, such as dividing alignments by gene and codon position. Because our method is data-driven, it can be used to estimate partitioned models for all types of alignments, including those that are not amenable to traditional approaches to partitioning.

## Background

The accuracy of phylogenetic inference often relies on the use of an appropriate model of molecular evolution [1,2]. Inaccurate tree reconstructions can be the result of both stochastic and systematic error. Stochastic error is the inevitable consequence of using finite datasets, and decreases as datasets grow in size. Systematic error results from biases such as the failure to adequately model the patterns of molecular evolution that generated the data (model misspecification) [3-5], and can be amplified in large datasets, often resulting in strong support for the incorrect tree topologies [3,6-10]. Improving approaches to model selection, even within existing phylogenetic frameworks, can help to reduce systematic error and improve the reliability of phylogenetic inference.

Accounting for the heterogeneity in the rates and patterns of evolution among sites in a DNA sequence alignment is an important part of selecting a model of molecular evolution [11-15]. Among the methods proposed to account for this are mixture models [16-18] and partitioning [19-22]. Mixture models account for among-site heterogeneity by combining estimates of the likelihood of each site in the alignment under more than one model of molecular evolution. Partitioning accounts for among-site heterogeneity by splitting an alignment into several groups of sites (subsets) and estimating model parameters independently for each subset. Although mixture models are an elegant way to account for among-site heterogeneity, partitioning remains more popular, more widely implemented, and is currently the only approach that is computationally efficient enough to work on very large datasets [23-29]. Thus, our focus in this manuscript is on developing methods to improve the selection of partitioning schemes for phylogenetic analyses, with a view to improving the inference of phylogenetic trees from large datasets.

An inherent obstacle in partitioned phylogenetic analyses is the choice of an appropriate partitioning scheme. One approach would be to evaluate every possible partitioning scheme for a given dataset and choose the best scheme, perhaps according to one of the commonly used information theoretic metrics such as the AICc [30] or BIC [31] or by some measure of biological features in the data. However, comparing all possible partitioning schemes is practically impossible because the number of partitioning schemes is astronomical even for very small alignments [32,33]. For example, some of the smallest alignments used today, associated with DNA barcoding studies, contain ~658 base pairs [34], which can be grouped into more than 1.0 × 10^931^ possible partitioning schemes: well beyond anything that can be feasibly analyzed by brute force. A related approach is to allow the data inform the assignment of sites to subsets, and to integrate out the uncertainty in these assignments in a Bayesian framework [35]. Although this method is elegant, it has a high computational burden that renders it impractical for all but modestly sized datasets.

The most commonly used method for partitioning alignments, and the only one currently suited to very large datasets, is to define subsets according to structural features of the sequences in the alignment, such as gene boundaries, codon positions, structural components of rRNAs (such as stems and loops), or some combination of these. We call this ‘traditional’ partitioning throughout this manuscript. This approach is also known as mechanistic modeling because it describes known biological or mechanistic processes and is motivated by the observation that different molecular features can have different patterns of molecular evolution [20,21,36-43]. Recently, various methods have been proposed to algorithmically refine traditional partitioning schemes by grouping together similar subsets of sites [29,32,33]. One example of this method is the PartitionFinder greedy algorithm [33], which works by joining a pre-defined subset with every other pre-defined subset and then selecting the grouping that most improves the AICc or BIC score. This is repeated until no more groupings improve the score. Using this method can result in large improvements in model fit. However, despite their popularity, all traditional partitioning approaches make an important assumption that is rarely questioned: that all of the sites in each of the pre-defined subsets (e.g. a particular codon position in a particular gene) have evolved under a single evolutionary model.

A number of recent studies have suggested that traditional approaches to partitioning can be inadequate. Evidence suggests that there can be substantial heterogeneity of the evolutionary process within a single codon position of a single gene [22,44-48] and within a single stem or loop of rRNA [16,46,49,50]. If this is true, then traditional approaches to partitioning may fail to adequately account for the variation in patterns of molecular evolution within each traditionally defined subset of sites. For smaller datasets, these limitations can be overcome by applying newer methods [18,35], but for larger datasets the limitations of traditional partitioning remain a problem.

Another limitation of traditional partitioning involves its application to new types of molecular markers. Many of the latest methods for assembling phylogenomic datasets result in large alignments that consist either entirely or largely of non-protein coding DNA (e.g. introns and ultra-conserved elements (UCEs)) [51-54]. It can be difficult to determine a good partitioning scheme for these datasets with traditional approaches because we understand little about the molecular evolution of the sequenced regions, and the datasets lack convenient features such as codon positions on which subsets can be defined *a priori*. Thus, we face the problem that we lack adequate ways to model molecular evolution for some of the largest and most promising empirical datasets in our field.

One approach to choosing a partitioning scheme for large datasets is to group sites into subsets using estimates of site rates [22,55-57]. Kjer and Honeycutt [22] showed that partitioning an alignment in this way resulted in a mammal mitochondrial genome phylogeny that was better supported and more congruent with phylogenies based on nuclear data. Ellingson et al. [56] showed that this approach improved both topologies and node support for a phylogeny of fish. However despite their promise, these methods have not been widely adopted. This is perhaps because they are difficult to use and require various decisions (such as the appropriate number of subsets into which to divide the data) to be made before the analysis is conducted.

In this study, we develop a new algorithm that automatically defines partitioning schemes by clustering similar sites together into subsets. Our approach improves on previous work in three important ways. First, while previous approaches [22,55-58] have required the user to choose the number of subsets before the analysis is carried out, our method estimates the optimal number of subsets directly from the data. This is important, because the optimal number of subsets may be difficult to predict in advance, and is influenced by several variables: e.g. the variation in substitution patterns among sites, the range of GTR submodels that can be selected for each subset, and the method used to evaluate the fit of the model to the subset (e.g. AICc, BIC). Second, our method scales to work with the large datasets being produced today. Third, we explicitly test for, and address, the presence of a suspected bias in previous implementations of this approach: that the partitioning scheme selected by the method may be biased towards the phylogenetic tree from which the site rates were calculated [55].

We demonstrate our approach on a wide range of datasets. Our results show that our method can be used to select partitioning schemes for the full range of datasets used in phylogenetics: from small barcoding datasets to large phylogenomic datasets consisting of ultra-conserved elements. In all cases, our method finds partitioning schemes that outperform those selected with traditional approaches to partitioning, when measured by metrics such as AICc and BIC.

## Methods

### Terminology

We follow the terminology established in other studies on partitioning [29,32,33] where a ‘subset’ refers to a set of sites for which the parameters of a nucleotide substitution model are estimated independently from other subsets. Each site can be assigned to only one subset. In the phylogenetics community, a subset is often referred to as a ‘partition’; we avoid using the word ‘partition’ because it has conflicting definitions in other fields [29,33]. A ‘partitioning scheme’ constitutes a collection of subsets that include every site in the alignment once and only once.

### Iterative *k*-means partitioning algorithm

**Figure 1:**
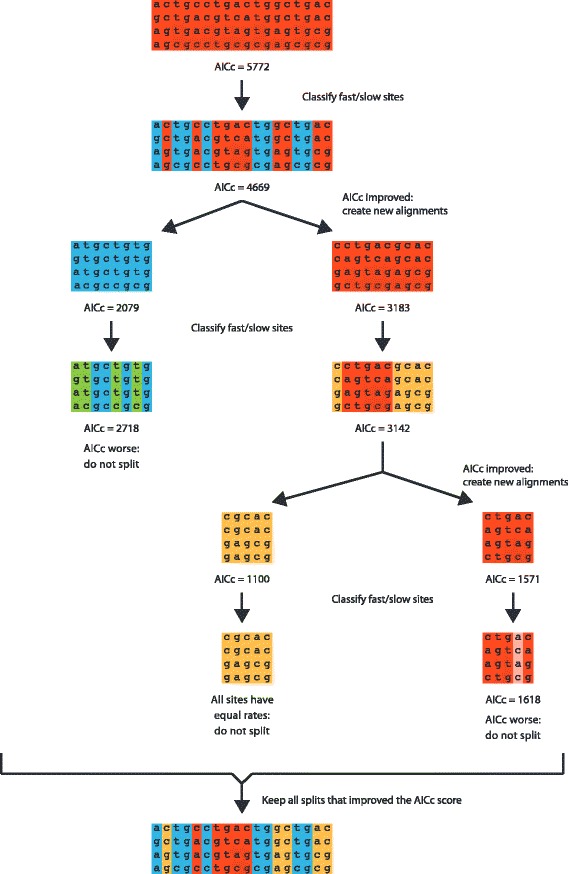
**This figure illustrates the progress of a hypothetical run of the iterative** ***k*** **-means algorithm.** The algorithm commences with an alignment that is treated as a single subset, and for which the AICc score has been calculated (step 3 in the description in the main text; represented by the red sequence alignment at the top). During this step, each of 56 GTR + I + G submodels is fit to the alignment and the model that returns the best AICc score is chosen. Next, the algorithm calculates TIGER site rates for each site (step 4a in the description in the main text), and uses these rates to classify the sites of the alignment into fast (red) and slow (blue) sites using the *k*-means algorithm (step 4b in the description in the main text). The AICc score of a model in which these two subsets are treated independently is then calculated (steps 4c-d in the description in the main text). If the score improves, the split is accepted. The fast (red) and slow (blue) sites are then used to create two new alignments, and the process is repeated with each new subset. This continues until no more subset splits are accepted. The final step combines all splits that improved the AICc score to create a single partitioning scheme for the dataset.

We present an algorithm (Figure 1) that automatically selects a partitioning scheme for a given alignment without the need for pre-defined subsets. We first give an overview, then expand on each step below:

1. Estimate a starting tree topology from the multiple sequence alignment;
2. Start with a partitioning scheme that has all sites assigned to a single subset, and choose the best-fit substitution model for that subset;
3. Calculate the information theoretic score of the current partitioning scheme;
4. For each subset in the current partitioning scheme, test whether that subset should be further divided:
  - Generate site rates for the focal subset;
  - Divide the focal subset into two subsets using *k*-means clustering;
  - Choose the best-fit substitution model for each of the two new subsets;
  - Calculate the information-theoretic score of the partitioning scheme in which the two new subsets from 4c replace the focal subset;
  - If the information theoretic score improves, label the focal subset for division.
5. If no subsets have been labeled for division, terminate the algorithm. Otherwise, define a new partitioning scheme in which each labeled subset in the list is replaced by the two correspondingly smaller subsets defined in step 4b and return to step 4.

In step 1, we estimate a tree topology with branch lengths for the dataset. Optimizing the tree topology at each step would be computationally intensive, particularly for large phylogenomic datasets. For this reason, we use a fixed tree topology throughout the course of the algorithm. In principle, any method to estimate a starting tree could be used since it has been argued that a non-random tree is likely to be sufficient for model selection [59-61]; in our implementation, we use the BioNJ algorithm implemented in PhyML [24] to estimate a neighbor joining starting tree, then re-optimize the branch lengths of this tree in PhyML using the GTR + I + G model.

In step 2, we define a partitioning scheme in which all sites in the alignment are assigned to a single subset, and we then select a best-fit model of molecular evolution for this subset. The model selection step uses an information theoretic metric (e.g. the AICc or the BIC) to choose a substitution model from a list of candidate models. Here we select the best model from the set of 56 submodels of the GTR model available in PartitionFinder v1.1.1 [29]. These include the GTR model and some of the most popular submodels implemented in PhyML, along with the model extensions using discrete gamma distributed site rates (+G) and/or a proportion of invariant sites (+I). During the model selection step, PartitionFinder provides two options for estimating branch lengths: ‘linked’ or ‘unlinked’. When the branch lengths are ‘unlinked’, all branch lengths are re-estimated for each model in the list. When branch lengths are ‘linked’, the relative branch lengths are determined by the tree estimated in step 1, and each model is afforded a single rate multiplier which can stretch or shrink all branch lengths in tandem. Although ‘unlinked’ branch lengths allow users to better account for heterotachy (variation in relative branch lengths among subsets), in practice, they add so many parameters to the overall substitution that they are rarely preferred. For that reason, in what follows, we use ‘linked’ branch lengths in all of our analyses, although the option to use ‘unlinked’ branch lengths remains.

In step 3 we calculate one of two information theoretic scores (the AICc and the BIC [33]) for the current partitioning scheme. At the start of the algorithm, when all sites are assigned to a single subset, this score is equal to that of the initial best-fit substitution model.

In step 4, we decide whether to subdivide each of the subsets in the current partitioning scheme. In step 4a, we fit a GTR + G model of molecular evolution to the subset, conditioned on the tree and its relative branch lengths estimated during step 1 using maximum likelihood in PhyML [24]. Then we use one of two methods to calculate site-specific rates for each site in the subset, (i) likelihood-based site rates or (ii) Tree Independent Generation of Evolutionary Rates (TIGER) site rates [57]. Likelihood site-rates depend on the branch lengths and therefore have to be recomputed for each new subset. Similarly, TIGER rates depend on the composition of site patterns in subsets so also have to be recomputed for each new subset. Likelihood site-rates are estimated in PhyML using the “--print_site_lnl” option. TIGER site rates are calculated using a non-tree based method that estimates the similarity among site patterns as a surrogate for evolutionary rates [57]. This method relies on the construction and comparison of set partitions for each alignment pattern. For example, if a given alignment pattern is “AACGGA”, the resulting set partition would be *P*(*i*) = {{1, 2, 6}, {3}, {4, 5}}*. P*(*i*) (the set partition for alignment pattern *i*) consists of a set of at most four sets, that contain the sequence numbers in the alignment pattern that have, respectively, the nucleotides A, C, G, or T at site *i.* The number of non empty sets, which we denote by |*P*(*i*)| is equal to the number of different nucleotides found in site pattern *i*. The character partition of each site is then compared to the character partition of every other site. The sites are evaluated for agreement with every other site using a “partition agreement score”, (*pa*(*i*, *j*)), which is defined as:

$$ pa\left(i,j\right)=\frac{{\displaystyle {\sum}_{x\in P(j)}a\left(x,P(i)\right)}}{\left|P(j)\right|} $$

where *a*(*x*, *P*(*i*)) is equal to 0 or 1 depending on whether *x* is compatible with the character partition of site *i*, i.e. if *x* is a subset of one of the sets in *P*(*i*):

$$ a\left(x,P(i)\right)=\left\{\begin{array}{c}\hfill 1\  if\ x\subseteq A\ for\  some\ A\in P(i)\hfill \\ {}\hfill 0\hfill \end{array}\right. $$

The rate (*r*_*i*_) for the alignment pattern at site *i* is then obtained by computing the mean partition agreement score across all sites:

$$ {r}_i=\frac{{\displaystyle {\sum}_{j\ne i}}pa\left(i,j\right)}{n-1} $$

where n is equal to the number of sites in the alignment. The sites that are more similar to the pool of sites in the alignment are considered slow with rates approaching 1.0 (invariant sites always return a rate of 1.0), while the sites that are less similar to the pool of sites in the alignment are considered fast with rates approaching 0. It is important to note that this method does not take into account the character state in an alignment pattern when the set partitions are compared, e.g. the set partition and resulting site rate of “AACGGA” would be identical to that of “TTGAAT”. Although software exists to calculate TIGER site rates [57], we found the existing implementation to be too slow to be useful. Instead, TIGER site rates are calculated using a fast, C++ based program that we developed [62].

In step 4b, we use the *k*-means clustering algorithm to divide the sites in the focal subset into two clusters based on one or more of the site-wise parameter estimates from step 4a. *K*-means is a fast clustering algorithm capable of handling large datasets with high dimensionality [63,64]. It clusters data points by minimizing the within-cluster sum of squares measured between each data point and its closest cluster ‘centroid’. The goal of *k*-means is to minimize the function:

$$ \underset{\left\{{\mu}_1,\dots, {\mu}_k\right\}}{ \min }{\displaystyle \sum_{h=1}^k}{\displaystyle \sum_{x\in {\chi}_h}}\left|\right|x-{\mu}_h\left|\right|{}^2 $$

Where *k* is the number of clusters, *μ* is the cluster centroid, and *x* is any given data point, in the case of this study, the site rates, and ‖*x*‖ is the *L*_2_ norm, or Euclidean length, of *x*. The algorithm proceeds through two steps:

1. The assignment step, in which each point is assigned to a cluster with its closest centroid.
2. The update step, in which cluster centroids are moved to the center (mean) of their new clusters.

The number of clusters (*k*) is chosen *a priori* and fixed at 2 in our case, and then *k* centroids are placed within the sample space. The initial placement of centroids is an important step; poor placement can result in an unsatisfactory exploration of the sample space and, although the algorithm may converge, it may only reach a local optimum. To avoid this, we use the *k-*means++ centroid initialization method, which has been shown to be superior when compared to other centroid seeding techniques such as random placement [65,66]. We perform 100 initializations of the *k*-means algorithm, selecting the initialization that best minimizes the within-cluster sum of squares. Following initialization, Euclidean distances between each data point and the centroids are calculated and each data point is assigned to a cluster based on its nearest centroid. The centroids are then moved to the mean of their respective clusters (the *k-*mean) and distances are recalculated. This process is repeated until the centroids no longer move beyond a threshold at the end of the iteration. We used the *k*-means algorithm from the scikit-learn package implemented in Python [67]. In theory, any statistic that can be estimated on a site-specific basis could be used for clustering. In what follows, we compare the performance of likelihood site rates and TIGER site rates.

In steps 4c and 4d, we use the output of the *k*-means algorithm to create two new subsets, and then use an information-theoretic metric to decide whether splitting the focal subset improves the overall model of molecular evolution. To do that, we first (step 4c) estimate the best model for each of the two new subsets from our set of candidate models as described above. We then (step 4d) calculate the information-theoretic score of two partitioning schemes: one in which the focal subset is retained as a single subset, and one in which the focal subset is divided into two new subsets. If the overall information theoretic score of the latter partitioning scheme is better, we label the focal subset as one that should be divided.

Once step 4 has been applied to all of the subsets in the current partitioning scheme, we ask whether there are any subset divisions that improved the overall information theoretic score (step 5). If there are none, then the algorithm terminates, since we are unable to find a partitioning scheme better than the current scheme. Otherwise, we divide all of the subsets that are labeled for division in step 4. Then the algorithm iterates.

### Pragmatic considerations

The algorithm above makes the assumption that likelihoods can be calculated for any collection of sites in an alignment. During the development of the algorithm, we found some cases in which PhyML was unable to analyze some subsets. This was usually because the alignments were too small or contained only sites with identical site patterns. Since our aim is to produce partitioning schemes that can be used to estimate phylogenetic trees with programs like PhyML, and since these problematic subsets are likely to occur during any approach similar to the one we describe here, we designed the following solution. First, we flag the problematic subsets as the algorithm proceeds, and make the conservative assumption that their site-likelihoods will be identical to their site-likelihoods in the larger subset from which they were generated. This allows us to estimate conservative information theoretic scores for partitioning schemes as the algorithm proceeds. At the end of the algorithm (i.e. after step 5), we combine each of the problematic subsets with their nearest neighbor subset, defined as the non-problematic subset with the centroid (estimated in step 3a) that has the shortest Euclidean distance to the centroid of the problematic subset. This process is repeated until there are no problematic subsets, i.e. until PhyML can successfully analyze all of the subsets in the partitioning scheme.

### Empirical evaluation

To evaluate the performance of the iterative *k*-means algorithm, we compared ten partitioning scheme selection approaches on ten different datasets (Table 1). The approaches comprise five different partitioning methods, each of which was applied with both the BIC and AICc (Table 2). The five methods we compared were: (i) no partitioning (i.e. treating all sites as belonging to a single subset); (ii) partitioning by gene and codon position/rDNA stems and loops (all); (iii) optimizing the partitioning scheme from (ii) using the greedy algorithm implemented in PartitionFinder 1.1.1; (iv) iterative *k*-means with likelihood site-rates; (v) iterative *k*-means with TIGER site-rates.

**Table 1:** **Names, references, and clade information for the datasets used in empirical analyses**

**Dataset name**	**Clade (latin)**	**Clade (common)**	**Paper reference**	**Dataset reference**
Anderson 2014	Cephalopoda: Loliginidae	Pencil squids	[84]	[85]
Cognato 2001	Coleoptera: Scolytinae	Bark beetles	[86]	[87]
Grande 2013	Paracanthopterygii	Paracanthopterygian fish	[88]	[89]
Kang 2013a	Xiphophorus	Swordtail fish	[90]	N/A
Kawahara 2013	Hyposmocoma	Hawaiian fancy-cased caterpillar	[74]	[91]
Kjer 2007	Mammalia	Mammals	[22]	N/A
Leavitt 2013	Acridoidea	Grasshoppers	[36]	N/A
McCormack 2013	Neoaves	Birds	[68]	[82]
Oaks 2011	Crocodylia	Crocodilians	[92]	[93]
Sharanowski 2011	Braconidae	Parisitoid wasps	[94]	[95]

**Table 2:** **AICc and BIC scores for every partitioning scheme selected for each dataset**

**Dataset name**	**No partitioning**	**User**	**Greedy**	**TIGER site rates**	**Likelihood site rates**
AICc					
Anderson 2014	36721	34972	34972	32584	28681
Cognato 2001	39373	37172	37172	36262	32216
Grande 2013	111819	108501	108501	105300	96616
Kang 2013	34157	33699	33682	30091	19490
Kawahara 2013	32238	29940	29940	28385	23488
Kjer 2007	1120392	1075872	1075749	1059594	1008180
Leavitt 2013	445052	423826	423389	410220	371585
McCormack 2013	972461	963551	961143	828180	690645
Oaks 2011	80550	74814	74686	69278	58947
Sharanowski 2011	218188	213696	213696	211251	198268
BIC					
Anderson 2014	38444	36820	36820	34590	30649
Cognato 2001	39890	38067	38056	37168	32701
Grande 2013	112673	109862	109741	107929	100751
Kang 2013	34578	34650	34364	31617	30448
Kawahara 2013	33057	31052	30989	29561	25020
Kjer 2007	1121793	1081392	1079270	1065998	1020447
Leavitt 2013	445625	429638	425574	413343	379692
McCormack 2013	973122	992389	967479	830140	748946
Oaks 2011	81673	77190	76316	71117	58988
Sharanowski 2011	219937	215953	215872	214802	202707

During the empirical evaluation, one dataset, McCormack 2013 [68], was too large and partitioned into too many pre-defined subsets to analyze with PartitionFinder’s greedy algorithm in a reasonable amount of time. For this dataset, we used the relaxed clustering algorithm [69] in PartitionFinder 1.1.1. Relaxed clustering is optimized for large datasets and uses RAxML [70] for all likelihood calculations. Since only two nucleotide substitution models are implemented in RAxML (GTR + G and GTR + I + G) we used a two-step approach. First, the optimal partitioning scheme was selected using the relaxed clustering algorithm for the two RAxML models, and second, we reselected models for each subset of the initial partitioning scheme with the ‘user’ option in PartitionFinder 1.1.1, but this time with PhyML and considering the full set of models used in every other treatment. This allowed us to directly compare the information theoretic scores of this partitioning scheme with those selected by the other methods.

### Starting tree bias evaluation

Although it has been shown that a starting tree topology is unlikely to negatively affect model selection as long as it the starting tree is non-random [60], it was unclear whether this would be true for the iterative *k*-means method we develop here. Specifically, we were unsure whether site rates calculated under the assumption that the starting tree is true would bias our partitioning schemes toward recovering the starting tree during downstream phylogenetic analyses. Thus, we designed a simple test to evaluate starting tree bias.

To test whether the starting tree introduced bias into the estimation of the partitioning scheme, we used a five-step process. First, we estimated a neighbor-joining (NJ) tree for the data. Second, we created twenty new trees, where each new tree was a single subtree-prune and regraft (SPR) move away from the NJ tree, giving a set of 20 plausible non-random trees for the dataset. Third, we used these 20 trees as starting trees from which we estimated 20 partitioning schemes for each dataset using three methods: the PartitionFinder greedy algorithm, iterative *k*-means with likelihood site-rates, and iterative *k*-means with TIGER site rates (i.e. 60 partitioning schemes for each dataset, 600 in total). Fourth, we estimated a maximum-likelihood phylogenetic tree using RAxML for all 60 partitioning schemes for each dataset. For each of the three methods, the process resulted in a collection of 20 distinct starting trees, and 20 estimated ML trees. The final step in the process involved statistically testing whether the starting trees are more similar to their corresponding ML trees than would be expected by chance. To do this, we used a bootstrap test in which the observed test statistic is the sum of the Robinson-Foulds [71] distances between each starting tree and the corresponding ML tree (i.e. the ML tree estimated from the partitioning scheme that assumed the corresponding starting tree). For example, in the most extreme case, where each ML tree is identical to its corresponding starting tree, the observed test statistic would be zero. The null distribution of this test statistic is then estimated by re-calculating the test-statistic 999 times after randomly shuffling the list of ML trees each time. If the starting tree biases the estimation of the ML tree, then we expect the observed test statistic to be in the lower tail of the null distribution. We calculate the one-tailed p-value from the position of the observed test statistic in a ranked list of the values of the test statistic from the null distribution.

### Simulation example

While this paper was primarily aimed at evaluating the efficacy of the iterative *k*-means algorithm on empirical datasets, we also evaluated our method with a simple simulation. First, we simulated a tree under the Yule (pure-birth) process in INDELible v1.03 [72]. We chose a rooted tree and specified the following parameters for the simulation: number of tips-100, birth-0.1, and death-0 with a tree depth of 0.1. We then simulated a 1,000 bp alignment using the Jukes Cantor [73] model. Next, we scaled the tree from the first run to a tree depth of 1.0 (i.e. ten fold larger than the initial tree) and simulated another 1,000 bp alignment using Jukes Cantor. Finally, we concatenated the alignments (total: 2,000 bp) and estimated a partitioning scheme for it using iterative *k*-means with TIGER rates. Each step, from tree simulation through partitioning scheme selection was repeated 20 times. These conditions were chosen to explicitly test whether the iterative *k*-means algorithm would 1) assign alignment sites to subsets containing other sites generated from the same model, and 2) find the correct number of subsets.

## Results and discussion

The primary purpose of this study was to describe the iterative *k*-means algorithm and evaluate its performance on empirical data as compared to other commonly used partitioning strategies. To do this, we selected partitioning schemes for published empirical datasets using several different methods and compared the relative fit of each partitioning scheme using AICc and BIC.

**Figure 2:**
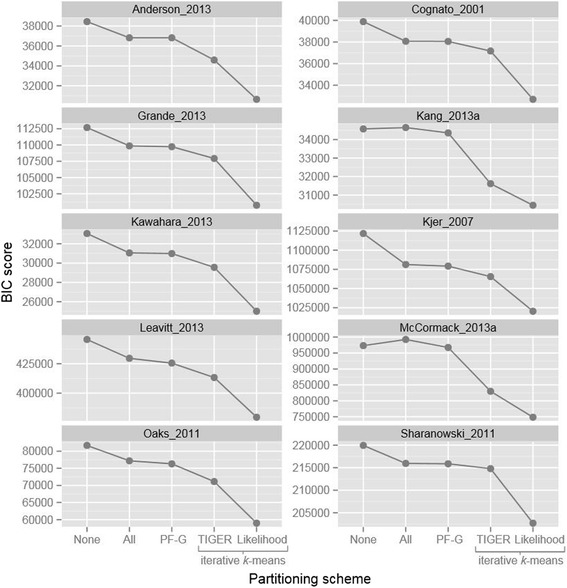
**BIC scores for partitioning schemes estimated during empirical testing (lower is better).** The *k*-means methods presented here outperform traditional methods. “None” is no partitioning, “All” is the user partitioning scheme, “PF-G” is the PartitionFinder greedy algorithm, “TIGER” is iterative *k*-means using TIGER site rates, “Likelihood” is iterative *k*-means using likelihood site rates. Note: The “PF-G” score for the McCormack 2013 dataset was obtained using the PartitionFinder relaxed clustering followed by model selection with PhyML as described in the Methods, not the greedy algorithm.

**Figure 3:**
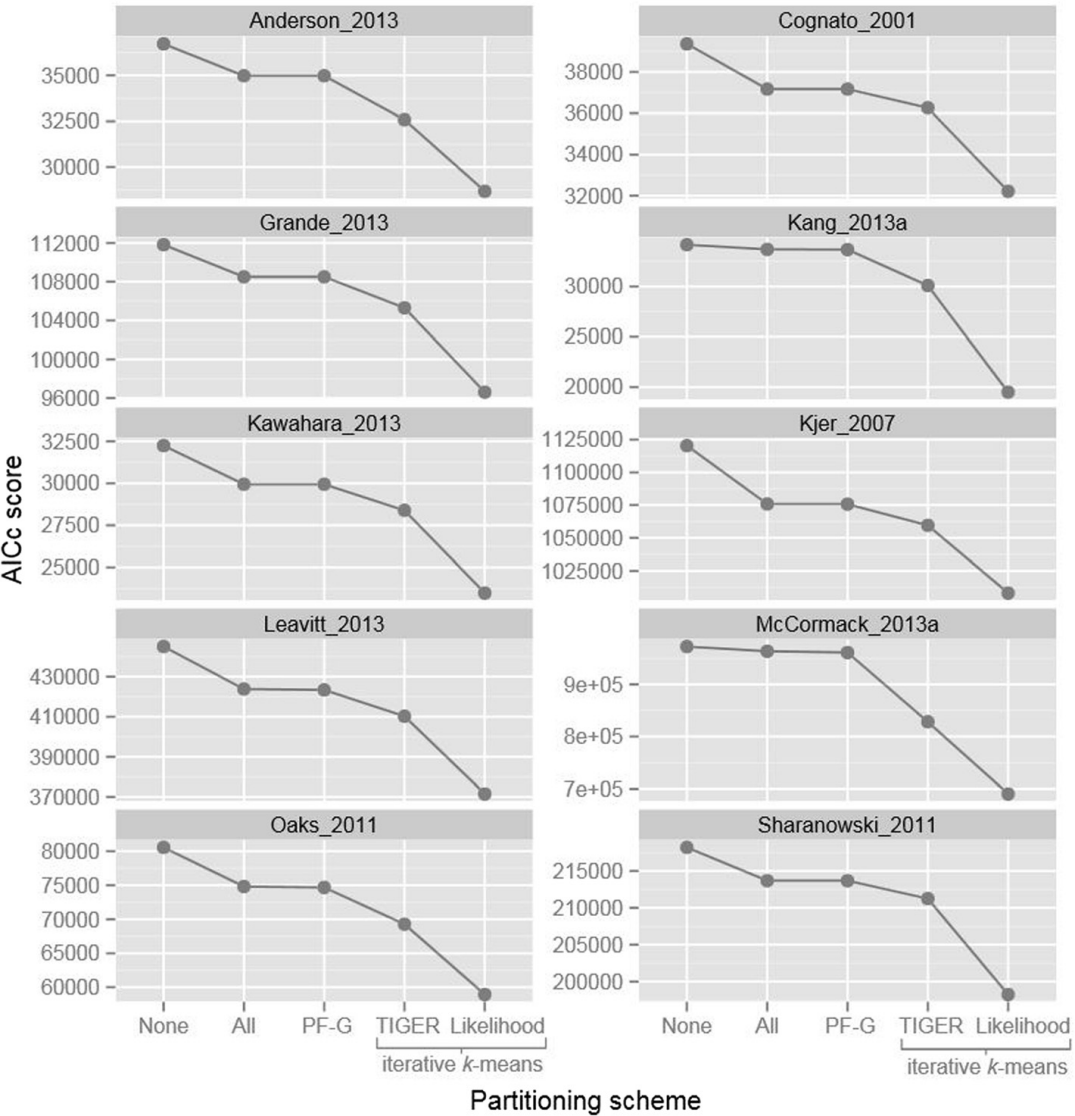
**AICc scores for partitioning schemes estimated during empirical testing (lower is better).** The *k*-means methods presented here outperform traditional methods. “None” is no partitioning, “All” is the user partitioning scheme, “PF-G” is the PartitionFinder greedy algorithm, “TIGER” is iterative *k*-means using TIGER site rates, “Likelihood” is iterative *k*-means using likelihood site rates. Note: The “PF” score for the McCormack 2013 dataset used the PartitionFinder relaxed clustering followed by model selection with PhyML as described in the Methods, not the greedy algorithm.

The iterative *k*-means algorithm substantially outperformed all other partitioning approaches for each of the ten datasets we analyzed, regardless of the details of the *k*-means approach or the information theoretic metric we used (Table 2, Figures 2 and 3). The set of alignments that we used to test the algorithms comprise a wide range of lengths, number of taxa, and types of molecular markers, confirming the utility of our new algorithm for a wide range of phylogenetic analyses.

Figures 2 and 3 show comparisons of the AICc and BIC scores achieved by five partitioning methods: using a single partition; partitioning according to structural features of the sequences; optimizing a partitioning scheme based on structural features using PartitionFinder; iterative *k*-means partitioning with likelihood-based site rates; and iterative *k*-means partitioning based on site rates estimated using the TIGER method. Figures 2 and 3 show that both *k*-means methods we describe here consistently outperform all of the other methods. The figures also suggest that the likelihood-based method is superior, as it consistently outperforms the method based on TIGER rates, achieving lower AICc and BIC scores. However, the apparent superiority of the likelihood-based method comes at a cost – it is also frequently associated with a bias: phylogenetic trees estimated from partitioning schemes derived from the likelihood-based approach were often more similar to the starting trees than would be expected by chance (Table 3). In 4 out of 9 datasets (Table 3), our test for starting tree bias returned a statistically significant result (p-value of < 0.05) for the likelihood-based method.

**Table 3:** **P-values and effect sizes for each dataset from starting tree bias analysis**

**Dataset**	**Partitioning method**	**p-value**	**effect size**
Anderson_2013	Greedy algorithm	1	0
Anderson_2013	lnL rates *k*-means	0.03	−0.604
Anderson_2013	TIGER rates *k*-means	1	0
Cognato_2001	Greedy algorithm	1	0
Cognato_2001	lnL rates *k*-means	0.006	−0.424
Cognato_2001	TIGER rates *k*-means	0.202	−0.152
Grande_2013	Greedy algorithm	1	0
Grande_2013	lnL rates *k*-means	0.047	−0.225
Grande_2013	TIGER rates *k*-means	1	0
Kang_2013a	Greedy algorithm	1	0
Kang_2013a	lnL rates *k*-means	0.391	−0.105
Kang_2013a	TIGER rates *k*-means	1	0.14
Kawahara_2013	Greedy algorithm	0.397	−0.095
Kawahara_2013	lnL rates *k*-means	0.008	−0.348
Kawahara_2013	TIGER rates *k*-means	0.828	0.051
Leavitt_2013	Greedy algorithm	1	0
Leavitt_2013	lnL rates *k*-means	1	0
Leavitt_2013	TIGER rates *k*-means	1	0
McCormack_2013a	Greedy algorithm	0.409	−0.116
McCormack_2013a	lnL rates *k*-means	0.52	−0.048
McCormack_2013a	TIGER rates *k*-means	0.158	−0.084
Oaks_2011	Greedy algorithm	1	0.094
Oaks_2011	lnL rates *k*-means	1	0.019
Oaks_2011	TIGER rates *k*-means	1	0
Sharanowski_2011	Greedy algorithm	1	0
Sharanowski_2011	lnL rates *k*-means	0.056	−0.304
Sharanowski_2011	TIGER rates *k*-means	0.069	−0.222

In contrast, when using the TIGER based rates we found no evidence for starting tree bias in any of the datasets that we examined. We attribute the difference between these two methods to the fact that the likelihood-based approach relies on a particular starting tree to calculate rates of evolution, whereas the TIGER method calculates rates without assuming a particular tree [57]. It appears that the dramatic gains in AICc and BIC scores achieved using the likelihood-based *k*-means approach are partially attributable to overfitting the partitioning scheme to the starting tree, and that this overfitting can then bias subsequent phylogenetic analyses. One symptom of this overfitting is that the likelihood-based rates method often selected subsets of sites that consisted entirely of invariant sites of a single nucleotide state. Such subsets are difficult if not impossible to justify on biological grounds. Together, these characteristics suggest that the likelihood method is problematic, and should be avoided. For the remainder of the paper, we focus only on the results from our study that used rates calculated with the TIGER method, which do not show these undesirable characteristics.

One of the primary motivations for this study was to develop a method to select partitioning schemes for datasets that are very large and/or that comprise molecular markers that are not amenable to traditional partitioning approaches, both of which are increasingly common [51,53,54]. It is encouraging, therefore, to note that the iterative *k*-means algorithm performed particularly well on the phylogenomic bird dataset (Table 2, Figures 2 and 3) [68], which was both very large and comprised solely of UCE’s, for which traditional approaches to partitioning are difficult to apply. For example, when each UCE was placed in its own subset, the BIC score was worse than when all UCE’s were grouped into a single subset (BIC scores of 992,389 and 973,121 respectively (Table 2)). When the partitioning scheme was selected using the relaxed clustering algorithm in PartitionFinder, the BIC score improved to 967,478 (Figure 2, Table 2), but when the partitioning scheme was selected using the iterative *k*-means method with TIGER rates, the BIC score improved to 830,140, a substantial improvement (Figure 2, Table 2).

**Figure 4:**
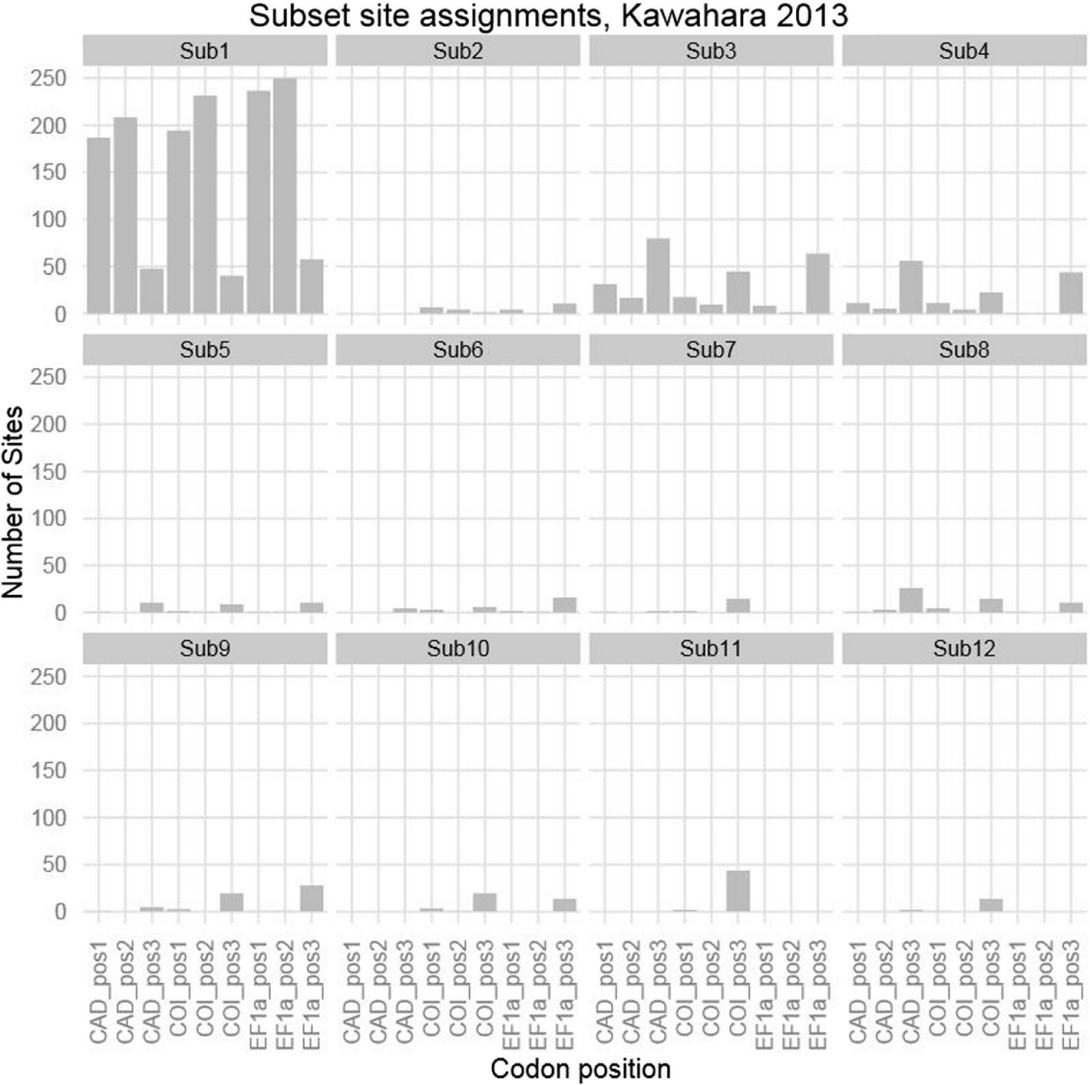
**Assignment of codon position by gene to subsets selected using the iterative** ***k*** **-means algorithm clustered using TIGER site rate estimates on the Kawahara 2013 dataset.** Subsets are ordered by the mean site rate from slowest to fastest. Sites from each codon position are spread throughout the subsets with the majority of variation among sites in the 3^rd^ codon position.

**Figure 5:**
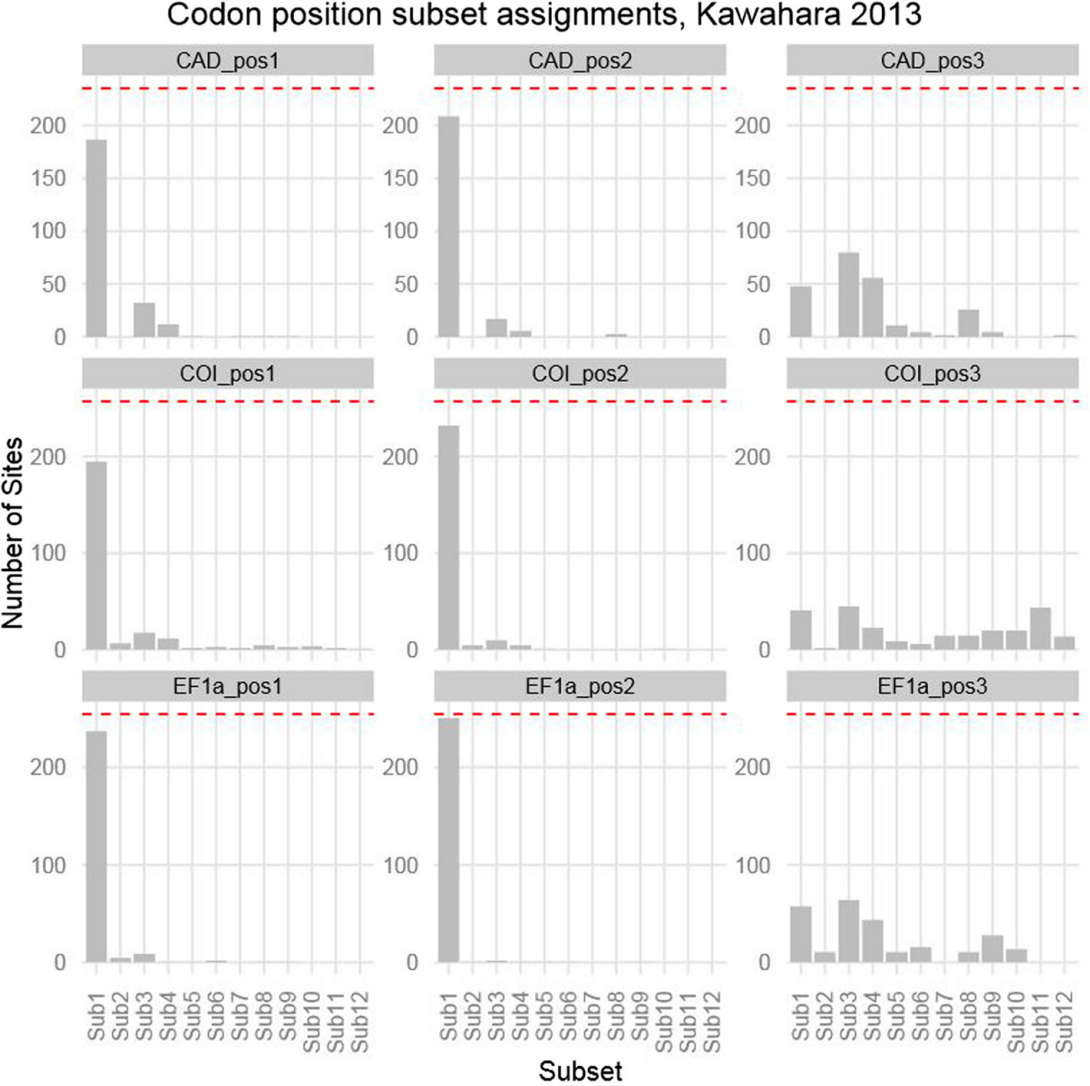
**Subset assignments for the sites from each codon position using the iterative** ***k*** **-means algorithm clustering using TIGER site rate estimates on the Kawahara 2013 dataset.** Each row corresponds to a single gene and each column corresponds to a different codon position. The dotted red line represents the total number of sites in each codon position. In each chart, subsets are ordered by the mean site rate from slowest to fastest. First and second codon positions most closely align with “traditional partitioning”, while substantial variation exists among the 3^rd^ codon position sites.

The iterative *k*-means clustering also worked well for datasets consisting of protein coding genes from the standard phylogenetic toolbox. A close examination of the partitioning schemes reveals that the algorithm chooses subsets that reflect the traditional biological partitioning boundaries such as genes and codon positions (Figures 4 and 5). For example, in the partitioning schemes selected for the Hawaiian fancy-case caterpillar dataset consisting of three protein-coding genes [74], the k-means approach resulted in one large subset that contained almost all first and second codon position sites across all three genes along with some third codon position sites (Figure 4, subset 1, Figure 5), and eleven smaller subsets which consisted primarily of third codon positions sites from the three loci (Figure 4, subsets 2–12, Figure 5). Insofar as it broadly combines first and second codon positions, and separates out third codon positions, this partitioning scheme is similar to a popular traditional partitioning method that does the same [75]. However, although some of the structure of the classical partitioning boundaries exists in the subsets chosen by our algorithm, other subsets include sites from a wide range of genes and codon positions (Figure 4 subsets 1–4, 6, 7). These results confirm that there is biological value to partitioning by genes and codon positions, but also suggest that relying solely on such boundaries may often fail to capture some of the complex patterns of molecular evolution among sites, potentially limiting the accuracy of downstream phylogenetic analyses.

**Figure 6:**
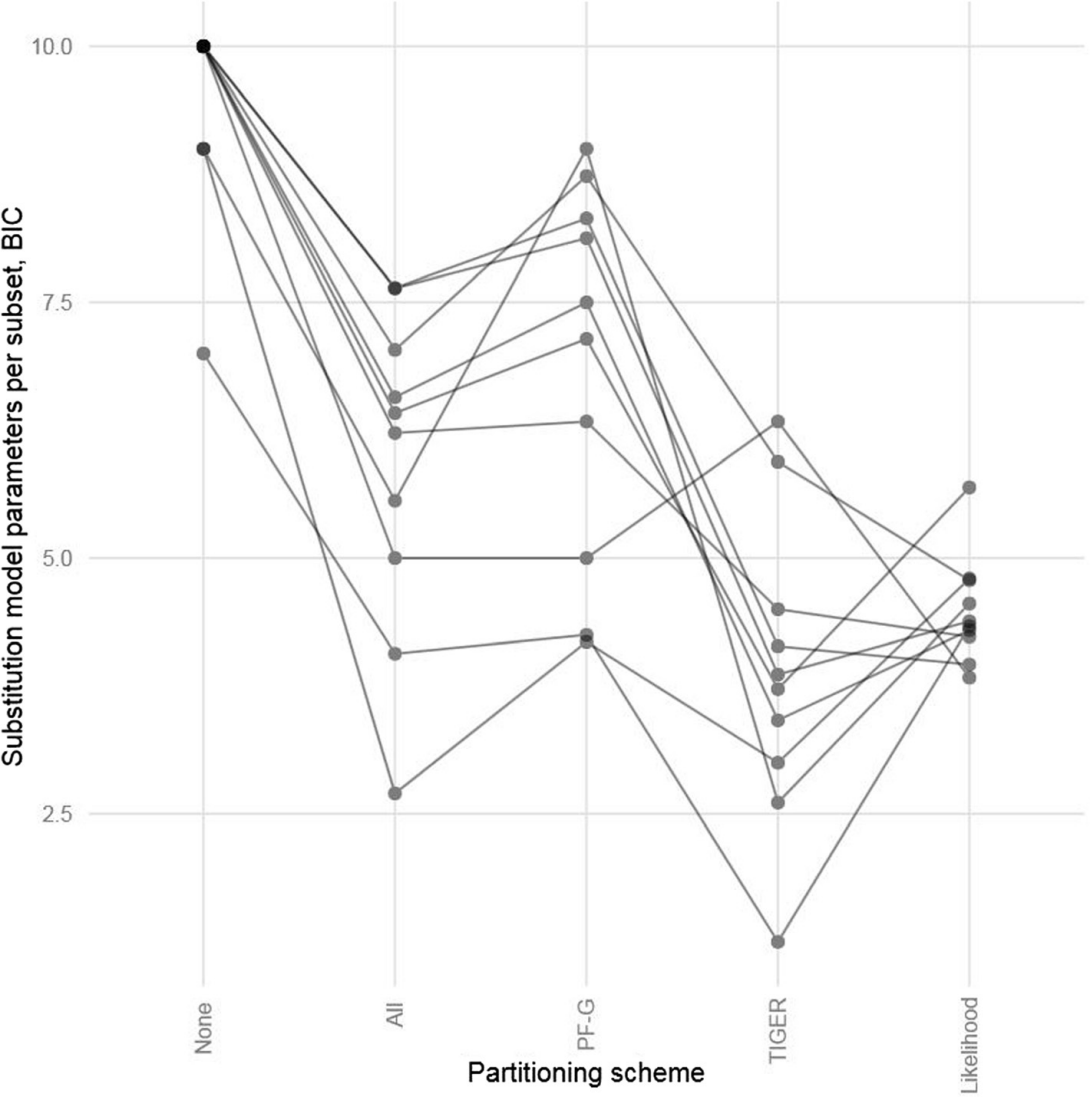
**Average number of parameters per subset for different partitioning scheme estimation methods using BIC.** Each line represents a different empirical dataset. “None” is no partitioning, “All” is the user partitioning scheme, “PF-G” is the PartitionFinder greedy algorithm, “TIGER” is iterative *k*-means using TIGER site rates, “Likelihood” is iterative *k*-means using likelihood site rates. The parameters per subset decrease for the *k*-means methods.

The iterative k-means algorithm provides a useful data-driven method to account for complex patterns of variation in rates of molecular evolution among sites. This is primarily because it tends to group together sites that evolve at similar rates of evolution, reducing the need for additional parameters to describe variation in rates across sites within a given group of sites (Figure 6). For example, in the crocodilian dataset [71], although 15 subsets were selected in the partitioning scheme chosen with TIGER rates for a dataset with just over 7,000 sites, models with the gamma model of rate heterogeneity were never chosen, and the proportion of invariable sites parameter was chosen for only three subsets. In contrast, the partitioning scheme chosen with the greedy algorithm included 11 subsets with seven that used either gamma or proportion of invariable sites in the model. Out of 168 total subsets selected using iterative *k*-means with TIGER rates and evaluated with BIC during our empirical evaluation, 77 (45.8%) required the additional parameters of gamma, proportion of invariable sites, or both. In contrast, of the 92 subsets chosen with the PartitionFinder greedy algorithm, 86 (93.5%) of the models included gamma, proportion of invariable sites, or both. These results support recent observations that more flexible models of variation in rates among sites tend to fit the data much better than those that rely on distributional assumptions [76,77], and suggest that the iterative *k*-means approach to partitioning may be particularly useful when the variation in rates across sites cannot be adequately modeled using a combination of traditional partitioning (e.g. using genes and codon positions) and gamma-distributed rates [22,77]. Other methods for accounting for this kind of heterogeneity exist and include the CAT model, implemented into the program Phylobayes [18,48,78-80] and a “spike and slab” model recently described by Wu et al. [35] that has been implemented into BEAST 2 [81]. Our method provides an alternative to these approaches.

**Figure 7:**
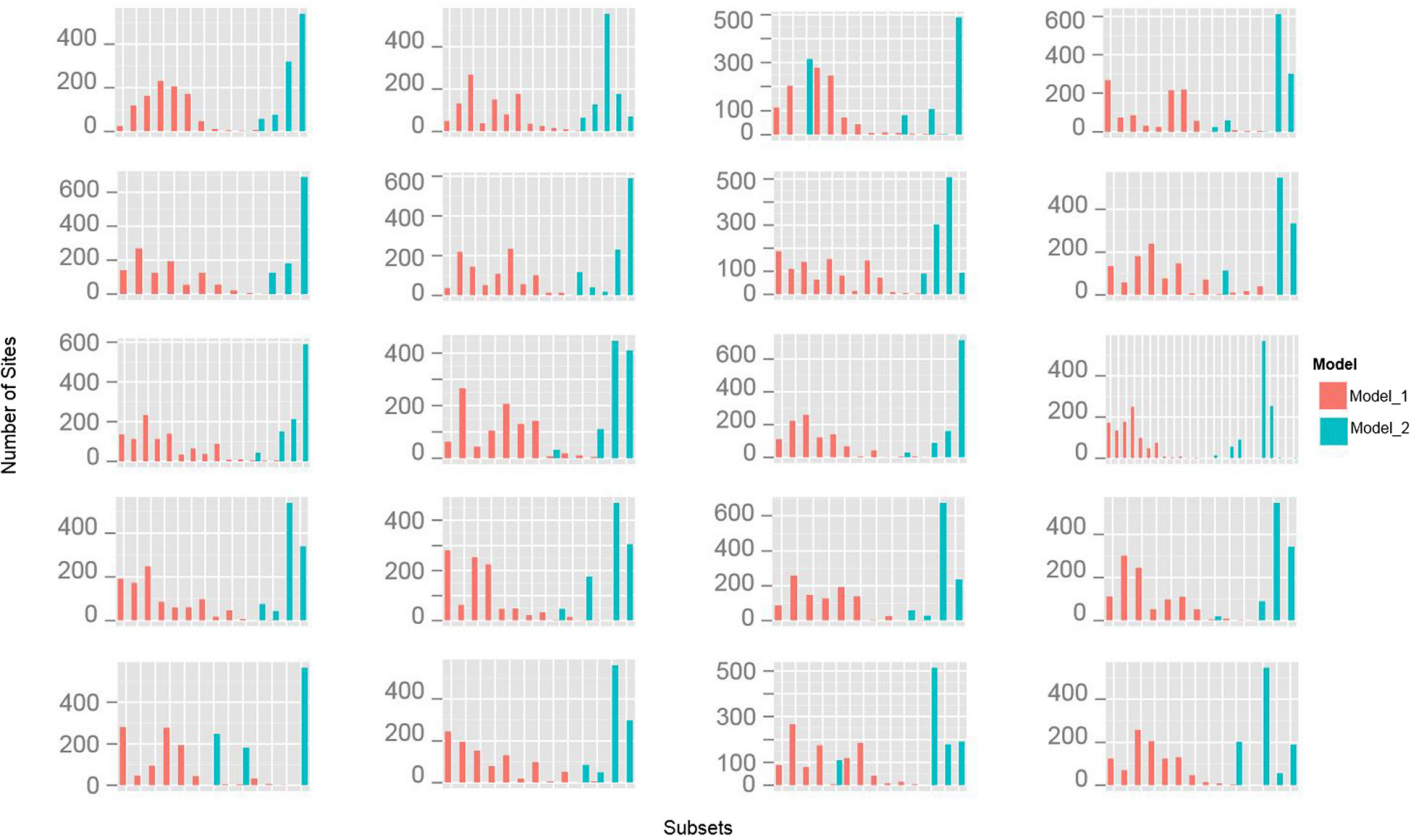
**Assignment of sites using iterative** ***k*** **-means and TIGER site rates for 20 simulated alignments.** The different colors represent sites generated under different models. The number of subsets selected is variable while sites are most often clustered with other sites simulated under the same model.

We evaluated the partitioning schemes chosen by the iterative *k*-means with TIGER rates for the simulated alignments based on the criteria that, 1) alignment sites generated under the same model would be assigned to the same subsets, and 2) the correct number of subsets would be chosen. Our results show that most subsets consisted primarily of sites generated from the same model (Figure 7). For example, in 263 out of 289 subsets (91%), at least 95% of the sites in the subset were generated under the same model (Table 4). However, the number of subsets varied from 12–24 (Table 4), far more than the two subsets under which the data were simulated. To further understand this behavior, we examined the partitioning schemes generated after each iteration of the algorithm. We found that the first split often closely approximated the true model, but due to continual increases in the BIC score, many more splits were accepted. This suggests that the inability to recover the true number of subsets could be due to the nature of the metrics for the evaluation of model fit. Whatever the underlying reason for the over-partitioning of simulated datasets, these results suggest that when using methods like these to select partitioning schemes for empirical studies, it would be prudent to estimate phylogenetic trees under a range of intermediate partitioning schemes as well as the final partitioning scheme. An important next step in investigating these and other approaches to partitioning is a full-scale simulation study which examines a broad range of simulation conditions, and which assesses the effects of each not only on recovering the correct model, but also on recovering the correct tree.

**Table 4:** **The number of subsets selected using the iterative** ***k*** **-means algorithm for 20 simulated alignments in which 2 independent subsets were simulated**

**Simulation replicate**	***k*** **-means subsets**	**Number of subsets consisting of > =95% sites from same model**
1	14	12
2	12	12
3	16	15
4	14	13
5	13	12
6	16	14
7	15	15
8	13	12
9	14	13
10	13	12
11	14	12
12	15	13
13	14	12
14	12	11
15	14	13
16	15	13
17	14	14
18	24	20
19	13	12
20	14	13

Despite the failure of the k-means method to recover the correct number of subsets in simulated data, three factors suggest that this finding is unlikely to severely compromise the method. First, previous studies have shown that defining too many partitions may have negligible impact on downstream phylogenetic inferences such as tree topologies, bootstrap support, or branch lengths [21,32]. Second, on empirical datasets, the *k*-means method tends to select a relatively modest number of subsets – never more than double the number of features in the dataset itself (e.g. individual codon positions in individual genes), and often many fewer. For example, for the McCormack et al. dataset [68,82], there were 416 individual UCE’s, and the k-means method selected just 18 subsets of sites. Third, the *k*-means method selects partitioning schemes that make biological sense with respect to what we already know about variation in rates and patterns of evolution (Figures 4 and 5).

It is important to note that the iterative *k*-means algorithm represents a heuristic search for an optimal partitioning scheme. As such, it cannot be guaranteed to find the optimum partitioning scheme for any given dataset. Furthermore, the *k*-means algorithm itself is somewhat stochastic in nature, and so it is likely that repeated analyses of the same dataset might lead to the estimation of partitioning schemes with very minor differences. Although we have focused on DNA sequence alignments in this study, the approach we describe can also be applied to amino acid alignments.

Our research suggests that the iterative *k*-means algorithm is an improvement over traditional approaches to partitioning. Accounting for variation of rates among sites has long been viewed as a vital part of modeling in phylogenetics [11,22,45,46,48,55,83], and we have shown that using site rates to inform subset assignments results in substantial improvements in the AICc and BIC scores of partitioning schemes, when compared to more commonly used methods. Perhaps most importantly, the iterative *k*-means algorithm provides a data driven method for modeling patterns of molecular evolution in markers such as UCE’s that have been difficult to model with traditional approaches.

## Conclusion

Partitioning remains the most commonly used method for accounting for variation in the rates and patterns of molecular evolution among sites in phylogenetic analyses. As the size and number of phylogenomic datasets grows, it is increasingly important to fit more realistic partitioned models to those datasets. The algorithm we present in this paper does this by automatically selecting a partitioning scheme for datasets of variable size and type without the need of an *a priori* determination of partition boundaries or number of desired subsets. Although we identified potential pitfalls of using such algorithms (such as a starting tree bias when using likelihood site rates), we also showed how these pitfalls could be overcome. These methods provide an important step forward in improving our approaches to modeling molecular evolution, particularly for very large datasets, as well as suggesting fruitful directions for further improvements.

### Availability of supporting data

All code, alignments, configuration input files, and output files have been uploaded to FigShare (http://dx.doi.org/10.6084/m9.figshare.1274798). The modified PartitionFinder code we developed for this manuscript is available at https://github.com/brettc/partitionfinder/tree/paul_develop and the TIGER rates code that we developed is available at http://dx.doi.org/10.5281/zenodo.12914. The data sets that were analyzed during empirical evaluation can be found at https://github.com/roblanf/PartitionedAlignments.

## Abbreviations

DNA: Deoxyribonucleic acid
rDNA: Ribosomal deoxyribonucleic acid
rRNA: Ribosomal ribonucleic acid
AIC: Akaike information criterion
AICc: Corrected Akaike information criterion
BIC: Bayesian information criterion
UCE: Ultra-conserved element
